# Association between niacin and mortality among patients with cancer in the NHANES retrospective cohort

**DOI:** 10.1186/s12885-022-10265-4

**Published:** 2022-11-14

**Authors:** Hongan Ying, Lijie Gao, Nansheng Liao, Xijuan Xu, Wenfeng Yu, Weiwen Hong

**Affiliations:** 1 Department of Geriatrics, Taizhou First People’s Hospital, Taizhou, 318020 P. R. China; 2 Department of Rehabilitation, Zhujiang Hospital of Southern Medical University, Guangzhou, 510000 P. R. China; 3 Department of General Surgery, Taizhou First People’s Hospital, Taizhou, 318020 P. R. China; 4 Department of Anus & Intestine Surgery, Taizhou First People’s Hospital, No. 218, Hengjie Road, Huangyan District, Taizhou, 318020 Zhejiang Province P. R. China

**Keywords:** Niacin, Mortality, Cancer, Retrospective cohort

## Abstract

**Background:**

The vitamin niacin is used as a lipid-regulating supplement, but it is unknown whether niacin has a positive influence on cancer prognosis. In this study, we examine the relationship between niacin intake and mortality among patients with cancer.

**Methods:**

Our study utilized all available continuous data from the National Health and Nutrition Examination Survey (NHANES) from 1999 to 2014. Multivariable Cox regression models were applied in order to investigate dietary niacin intake’s association with mortality. We compared the survival probability between groups of low and high niacin intake by plotting Kaplan-Meier curves. An analysis of subgroups was used to investigate heterogeneity sources.

**Results:**

A total of 3504 participants were included in the cohort, with 1054 deaths. One thousand eight hundred forty-seven participants (52.3%) were female, 2548 participants (73.4%) were white, and the mean age (SE) was 65.38 years (0.32). According to multivariate logistic regression analysis, niacin intake was negatively associated with mortality outcomes in patients with cancer, with *P* values below 0.05 in all models. In subgroup analyses based on sex, age, and BMI, the association persisted. The Kaplan-Meier curves indicate that high niacin intake groups have better survival rates than low intake groups. Niacin supplementation improved cancer mortality but not all-cause mortality.

**Conclusion:**

According to our study, higher dietary niacin intake was associated with lower mortality in cancer patients. Niacin supplements improved cancer survival rates, but not all causes of mortality.

**Supplementary Information:**

The online version contains supplementary material available at 10.1186/s12885-022-10265-4.

## Background

A diet plays a significant role in cardiovascular disease, cancer, and diabetes, which account for over 70% of global deaths [1]. Among these foods, fish, meat, milk, peanuts, and products made from enriched flour have high levels of niacin. Niacin (nicotinic acid or vitamin B3) is a functional group found in the coenzymes nicotinamide adenine dinucleotide (NAD) and nicotinamide adenine dinucleotide phosphate (NADP), which are essential for oxidative processes [2]. In pharmacological doses, niacin reduces blood lipids and increases highdensity lipoprotein (HDL). It was used as a vitamin supplement and to regulate lipid levels [3]. According to the results of two randomised controlled trials, adding niacin did not significantly reduce the risk of major vascular events among patients with atherosclerotic cardiovascular disease (CVD) [4, 5]. The use of niacin to prevent CVD is no longer recommended by clinical guidelines [6]. However, in the United States, niacin is still prescribed for other Food and Drug Administration (FDA) approved indications by thousands of patients [7]. Several recent large-scale clinical trials have found that niacin intake is associated with a reduced risk of squamous cell carcinoma (SCC) [8]. It might also protect against breast cancer recurrence and metastases [9]. Based on NHANES data, we investigated the association between dietary niacin and mortality in cancer patients.

## Methods

### Study population

Data from 8 cycles of the NHANES were used in this study (1999–2000, 2001–2002, 2003–2004, 2005–2006, 2007–2008, 2009–2010, 2011–2012, and 2013–2014).

The NHANES survey is a program that examines the effects of nutritional status on health promotion and disease prevention in the United States. The NHANES involves physical examinations and interviews. The interview includes questions about demographics, diet, socioeconomics, and health. The examinations include medical, dental, physiological, and laboratory tests etc. To identify a nationally representative sample of noninstitutionalized households, the NHANES used stratified, multistage recruitment. A nationally representative sample of approximately 5000 people is examined each year. Since 2007, data on vitamins, minerals, herbals, and other dietary supplements were included in the dietary interview. We excluded participants without dietary niacin data or with missing data on other covariates. Also excluded were those with insufficient identifying death data or not available for public release. Finally, 3504 participants were included in the analysis.

### Cancer status

In the NHANES, the medical conditions section provides self-reported health condition information [10]. Cancer diagnoses were based on the following two questions: 1. “ Have you ever been told by a doctor or other health professional that you had cancer or a malignancy of any kind? “; 2.“ What kind of cancer was it and when it was diagnosed? “. These questions were asked, in the home, by trained interviewers using the Computer-Assisted Personal Interviewing (CAPI) system [11]. The CAPI system is programmed with built-in consistency checks to reduce data entry errors.

### Niacin intake in the diet and supplements

Participants’ dietary niacin intake data is obtained in the dietary interview component [12]. The dietary interview component is used to estimate how much food and drink was consumed during the 24-hour period, and to estimate the energy, nutrient, and other composition of the food and beverage consumed. Supplemental niacin intake data is obtained in the dietary supplement component. Participants in the NHANES were asked whether they had taken any dietary supplements in the past 24-h. Those who reported supplement use were asked to provide product name, frequency, duration, and serving form information [13].

There are two 24-hour dietary recall interviews available for all NHANES examinees. The first dietary recall interview is collected in-person in the Mobile Examination Center (MEC) and the second interview is collected by telephone 3 to 10 days later.

Since too much supplement niacin data was missing in the second day recall, we used the first day interview data for estimates.

### Mortality

In 2015, the National Center for Health Statistics published Public-use Linked Mortality Files (LMF) for NHANES 1999–2014 [14]. The files include mortality status and underlying causes of death. Cause-specific death was determined using the 10th Revision of the International Classification of Diseases (ICD-10) [15]. In this study, persons who did not have sufficient identifying data or were not available for public release were excluded.

### Covariates

The first day of the diet interview was used to obtain information about macronutrient intake like total energy, fat, protein, carbohydrates, cholesterol, sugar and micronutrients like vitamin B1, vitamin B2. The household interview collected demographic information and lifestyle factors, including age, sex, race/ethnicity, education, income, smoking, and physical activity. At the Mobile Examination Center, alcohol intake, weight, and height were recorded. The consumption of alcohol was defined as having at least 12 alcohol drinks per year. Those who have never smoked or who have only smoked in their lives less than 100 cigarettes were classified as never smokers [16]. Body mass index (BMI) was calculated by dividing weight (kg) by height in square meters. The diagnosis of diabetes was based on self-reported doctor/health professional diagnosis, the use of any antidiabetic medication, or the presence of glycosylated hemoglobin in blood levels above 6.5%. Physical activity defined by at least one vigorous-intensity activity per week [17]. The following two questions were used to diagnose CVD among participants: 1. “ has a doctor or other health professional ever told you that you had coronary heart disease? “; 2. “ how old were you when you were first told you had coronary heart disease? “.

### Statistical analysis

According to the instructions for using NHANES data, we accounted for complex survey design factors, such as clustering, stratification, and dietary weight at day one of the survey [18]. Public-use Linked Mortality Files contain the follow-up time from the in-person interview survey, and were used to assess person-time to death [19]. For continuous variables, means and standard errors were presented, while counts and proportions (after weighting) were presented for categorical variables [20]. We used the Student t-test to compare continuous variables, and the Chi-square test to compare categorical ones. The independent association between niacin intake and cause-specific mortality was assessed through multivariate Cox regression analysis. As a sensitive analysis, different covariates adjusted models were analyzed using an extended Cox model approach. Kaplan-Meier survival curves and log-rank analyses were used to compare survival probabilities [21]. In addition, the stratified analysis was conducted on the basis of clinicopathological and lifestyle factors. The likelihood ratio test was used to calculate the *P* value for interactions. Statistical significance was defined as a 2-sided *P* value less than 0.05. All statistical analyses were conducted using the R software (version 3. 6. 3, http://www.R-project.org/).

## Results

### Study participants and baseline characteristics

**Fig. 1:**
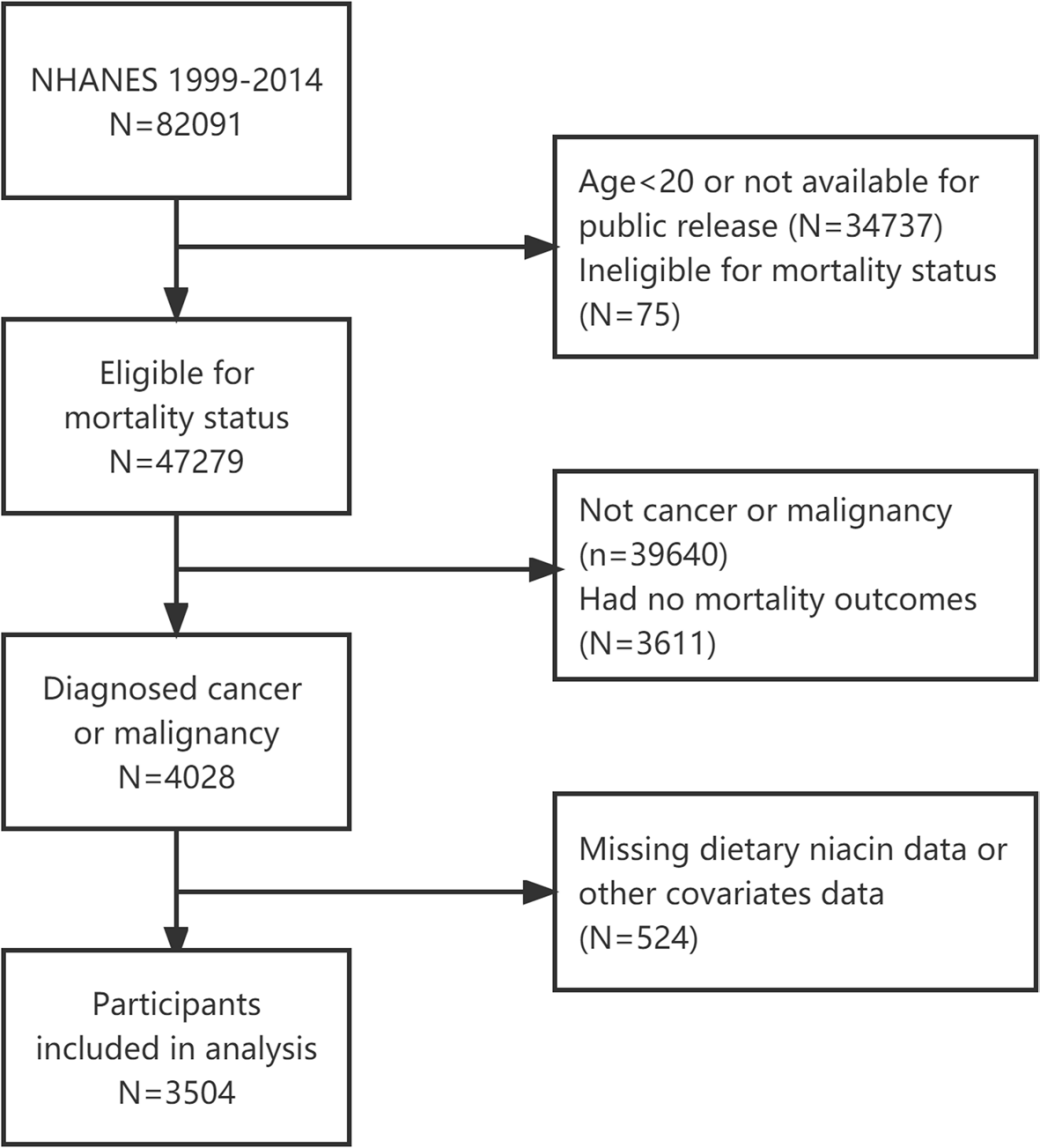
The flow chart of the study

A total of 82,091 individuals participated in eight consecutive NHANES 2-year cycles (1999–2014). The participants were followed up for mortality status until December 31, 2015, and 47,279 were eligible. We excluded participants who did not have dietary niacin data or other covariates and selected those diagnosed with cancer aged 20 years and older. In total, 3504 participants were studied, including 1986 with data on niacin supplementation (Fig. 1). Table 1 shows the baseline characteristics of the subjects by quartiles. The mean (SE) age of participants was 65.38 (0. 32) years, 1847 (52.3%) were female, 2548 (73.4%) were white, and 734 (30.8%) were non-Hispanic white individuals. Dietary niacin intake (mean (SE)) was 21. 82(0. 19) mg/day. Women, smokers, Mexican Americans, education less than high school graduation, and alcohol consumers were more likely to consume low niacin. Also were those with lower intakes of energy, sugar, fat, protein, cholesterol, fiber, as well as micronutrients like vitamins B1, B2.

**Table 1:** Baseline characteristics of participants stratified by dietary niacin intake

Variables	Total (*n* = 3504)	Quartile 1 (*n* = 876)	Quartile 2 (*n* = 876)	Quartile 3 (*n* = 876)	Quartile 4 (*n* = 876)	*P*-value
Sex n (%)						< 0.001
Male	1657 (47.7)	266 (30.6)	351 (40.2)	462 (52.6)	578 (67.0)	
Female	1847 (52.3)	610 (69.4)	525 (59.8)	414 (47.4)	298 (33.1)	
Age, Mean ± SE	65.38 ± 0.32	65.70 ± 0.55	65.81 ± 0.57	66.13 ± 0.50	66.88 ± 0.57	0.002
RACE, n (%)						0.893
Mexican American	230 (6.4)	78 (8.9)	61 (6.5)	47 (5.5)	44 (4.9)	
Other Hispanic	145 (4.0)	51 (5.1)	35 (4.2)	26 (2.9)	33 (3.8)	
Non-Hispanic White	2548 (73.4)	574 (66.8)	636 (72.9)	679 (77.9)	659 (75.9)	
Non-Hispanic Black	475 (13.3)	145 (16.4)	117 (13.3)	94 (10.4)	119 (13.2)	
Other Race - Including Multi-Racial	106 (2.8)	28 (2.7)	27 (3.1)	30 (3.2)	21 (2.2)	
Education, n (%)						< 0.001
Less than 9th grade	384 (10.9)	134 (15.2)	103 (12.0)	80 (9.1)	67 (7.5)	
9-11th grade	477 (13.1)	138 (15.3)	133 (14.4)	116 (13.0)	90 (9.8)	
High school graduate	823 (23.7)	215 (25.1)	215 (24.5)	206 (23.6)	187 (21.5)	
Some college or AA degree	958 (27.5)	239 (26.8)	225 (26.3)	236 (26.9)	258 (30.0)	
College graduate or above	860 (24.5)	148 (17.6)	200 (22.8)	238 (27.5)	274 (31.1)	
BMI, Mean ± SE	28.56 ± 0.10	28.59 ± 0.22	28.64 ± 0.22	28.72 ± 0.21	28.30 ± 0.23	0.494
Diabetes, n (%)						0.216
Yes	803 (23.3)	219 (25.3)	210 (24.1)	195 (22.7)	179 (21.1)	
No	2701 (76.7)	657 (74.7)	666 (75.9)	681 (77.3)	697 (78.9)	
Smoking, n (%)						0.281
Yes	1980 (56.8)	471 (53.5)	494 (57.0)	503 (57.4)	512 (59.2)	
No	1523 (43.2)	404 (46.5)	382 (43.0)	373 (42.6)	364 (40.8)	
Drinking, n (%)						< 0.001
Yes	2304 (68.5)	488 (58.3)	570 (67.5)	592 (70.4)	654 (77.5)	
No	1061 (31.5)	349 (41.7)	274 (32.5)	249 (29.6)	189 (22.5)	
Aspirin use, n (%)						0.248
Yes	471 (13.9)	106 (12.9)	115 (13.7)	135 (15.5)	115 (13.6)	
No	2964 (86.1)	749 (87.1)	750 (86.3)	731 (84.5)	734 (86.4)	
Vigorous activity, n (%)						0.002
Yes	545 (15.6)	116 (13.4)	123 (13.9)	129 (15.0)	177 (20.1)	
No	2959 (84.4)	760 (86.6)	753 (86.1)	747 (85.0)	699 (80.0)	
Energy intake (kcals/day), Mean ± SE	1889.41 ± 16.15	1277.11 ± 17.48	1674.28 ± 19.02	2005.15 ± 21.01	2589.15 ± 34.1	< 0.0001
Protein intake (g/day), Mean ± SE	72.27 ± 0.58	42.28 ± 0.63	61.74 ± 0.66	77.22 ± 0.72	107.23 ± 1.19	< 0.0001
Carbohydrate intake (g/day), Mean ± SE	232.72 ± 1.98	167.28 ± 2.76	208.61 ± 2.53	246.93 ± 3.18	306.80 ± 4.70	< 0.0001
Sugar intake (g/day), Mean ± SE	148.33 ± 7.49	107.35 ± 7.81	139.69 ± 12.64	155.87 ± 13.65	189.63 ± 15.06	< 0.0001
Fat intake (g/day), Mean ± SE	71.95 ± 0.78	47.64 ± 0.83	64.21 ± 1.06	76.61 ± 1.12	98.88 ± 1.83	< 0.0001
Cholesterol intake (mg/day), Mean ± SE	264.81 ± 4.08	190.66 ± 6.94	241.90 ± 6.38	267.36 ± 6.93	357.77 ± 8.64	< 0.0001
Fiber intake (g/day), Mean ± SE	15.99 ± 0.21	10.80 ± 0.23	14.22 ± 0.29	17.08 ± 0.34	21.77 ± 0.41	< 0.0001
Vitamin B1 intake(mg/day), Mean ± SE	1.53 ± 0.01	0.86 ± 0.01	1.29 ± 0.02	1.65 ± 0.02	2.30 ± 0.04	< 0.0001
Vitamin B2 intake (mg/day), Mean ± SE	2.06 ± 0.02	1.28 ± 0.03	1.74 ± 0.02	2.20 ± 0.03	3.01 ± 0.04	< 0.0001
Niacin intake (mg/day), Mean ± SE	21.82 ± 0.19	10.03 ± 0.10	16.80 ± 0.06	23.08 ± 0.07	37.12 ± 0.39	< 0.0001

Means and standard error were described for the continuous variables, counts and proportions (after weighted) were described for categorical variables

Abbreviations: *BMI* body mass index

### Dietary niacin intake and mortality outcomes

**Fig. 2:**
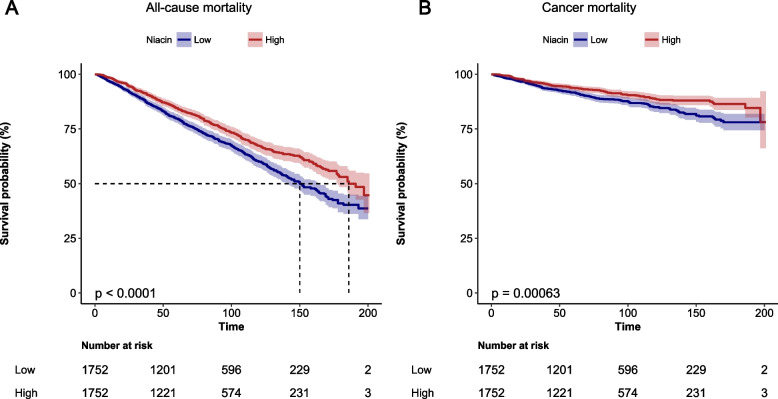
Kaplan-Meier Survival Curves for Mortality Outcomes. **A** for all-cause mortality, **B** for cancer mortality

We calculated the follow-up time using person months between the interview date and the date of death or the end of the mortality period. The 15-year follow-up documented 1054 deaths, including 342 cancer-related deaths (Table 2). After adjusting for other potential determinants, niacin intake was negatively correlated with mortality outcomes. Figure 2 demonstrates a statistically significant difference in survival probability between high and low niacin intake groups in mortality outcomes. In Non-adjusted Model, the HR for cancer mortality per 10 mg/day increase in niacin intake was 0.85 (95%CI: 0.77–0.95). In Model I, the HR was 0.83 (95% CI: 0.75, 0.93), while in Model II, the HR was 0.81 (95% CI: 0.67, 0.98) for the increase. For all-cause mortality per 10 mg/day increase, the HR was 0.88 (95% CI: 0.83, 0.94) in Non-adjusted Model, 0.87 (95% CI: 0.81, 0.92) in Model I, while 0.89 (95% CI: 0.80, 1.00) in Model II. The *P* values for all three models are below 0.05 for cancer mortality and all-cause mortality, respectively (Table 2). Compared to participants in the lowest quartile of niacin intake, those in the highest quartile had lower cancer mortality risks (Non-adjusted Model: HR = 0.61, 95%CI: 0.46–0.82; Model I: HR = 0.57; 95%CI: 0.42–0.77; Model II: HR = 0.51; 95%CI: 0.32–0.82). All-cause mortality risks were also lower among participants in the highest quartile of niacin intake compared to those in the lowest quartile (Non-adjusted Model: HR = 0.64; 95%CI: 0.54–0.77; Model I: HR = 0.61; 95%CI: 0.51–0.73; Model II: HR = 0.73; 95%CI: 0.55–0.97) (Table 2). There was a L-shaped relationship between dietary niacin and all-cause mortality (*P* for non-linearity =0.011). The benefit associated with increasing niacin intake achieved its maximum at approximately 25 mg/day and no further reduction in mortality was found beyond this level of intake. But in cancer mortality, we observed no apparent plateau (Supplementary Fig. 1).

**Table 2:** The relationship between dietary niacin intake and mortality among cancer patients, NHANES (1999–2014)

Outcomes	Non-adjusted Model		Model I		Model II	
	HR (95% CI)	***P***-value	HR (95% CI)	***P***-value	HR (95% CI)	***P***-value
**Cancer Mortality** No. of deaths/patients (342/3504)						
Dietary Niacin, 10 mg/day	0.85 (0.77–0.95)	0.003	0.83 (0.75–0.93)	0.001	0.81 (0.67–0.98)	0.032
**Niacin classification**						
Quartile 1	Reference		Reference		Reference	
Quartile 2	0.71 (0.53–0.93)	0.015	0.65 (0.49–0.86)	0.003	0.60 (0.44–0.82)	0.001
Quartile 3	0.56 (0.42–0.76)	< 0.001	0.52 (0.38–0.7)	< 0.001	0.49 (0.34–0.71)	< 0.001
Quartile 4	0.61 (0.46–0.82)	0.001	0.57 (0.42–0.77)	< 0.001	0.51 (0.32–0.82)	0.005
***P*** for Trend		< 0.001		< 0.001		0.001
**All-Cause of Mortality** No. of deaths/patients (1054/3504)						
Dietary Niacin, 10 mg/day	0.88 (0.83–0.94)	< 0.001	0.87 (0.81–0.92)	< 0.001	0.89 (0.8–1.0)	0.042
**Niacin classification**						
Quartile 1	Reference		Reference		Reference	
Quartile 2	0.86 (0.73–1.01)	0.067	0.81 (0.69–0.95)	0.011	0.81 (0.67–0.97)	0.021
Quartile 3	0.73 (0.61–0.86)	< 0.001	0.64 (0.54–0.76)	< 0.001	0.69 (0.55–0.85)	0.001
Quartile 4	0.64 (0.54–0.77)	< 0.001	0.61 (0.51–0.73)	< 0.001	0.73 (0.55–0.97)	0.027
***P*** for Trend		< 0.001		< 0.001		0.005

Model 1: Cox proportional hazards regression model stratified by age, sex, and race, bmi

Model 2: Further adjusted for education, smoking status, drinking,diabetes, aspirin use, physical activity, energy intake, protein intake, sugar, carbohydrate, total fat intake, Vit B1, VitB2, Cholesterol, fiber

Abbreviations: *HR* hazard ratio, *BMI* body mass index, *CI* confidence interval

### Sensitive analysis

Our conclusions were further supported by data in the dietary supplement component (NHANES 2007–2014). In the supplement group, total niacin intake is 76. 4 mg/day, while in the no supplement group, it is 21. 4 mg/day (Supplementary Fig. 2). Table 3 shows that niacin supplementation reduced cancer mortality (HRs range 0.48–0.58, *p* <  0. 05 for all models) but not all-cause mortality (HRs range 0.81–1.00, *p* > 0. 05 for all models).

**Table 3:** Dietary And Supplement Niacin Association With Mortality Among Patients With cancer NHANES (2007–2014)

Outcomes	Non-adjusted Model		Model I		Model II	
	HR (95% CI)	***P***-value	HR (95% CI)	***P***-value	HR (95% CI)	***P***-value
**Cancer Mortality** No. of deaths/patients(116/1986)						
No Supplement	Reference		Reference		Reference	
Supplement	0.58 (0.38–0.89)	0.012	0.50 (0.32–0.77)	0.002	0.48 (0.3–0.77)	0.002
**All-Cause Mortality** No. of deaths/patients(327/1986)						
No Supplement	Reference		Reference		Reference	
Supplement	1.00 (0.80–1.25)	0.991	0.81 (0.64–1.03)	0.083	0.92 (0.72–1.18)	0.530

Model 1: Cox proportional hazards regression model stratified by age, sex, and race, bmi

Model 2: Further adjusted for Further adjusted for education, smoking status, drinking, diabetes, aspirin use, physical activity, energy intake, protein intake, sugar, carbohydrate, total fat intake, Vit B1, VitB2, Cholesterol, fiber

Abbreviations: *HR* hazard ratio, *BMI* body mass index, *CI* confidence interval

### Dietary niacin intake and mortality within subgroups

**Fig. 3:**
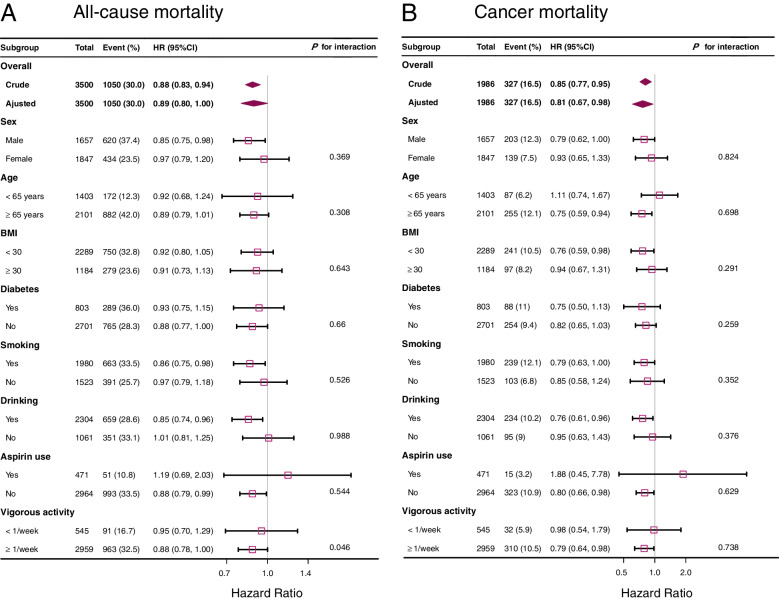
An Analysis of Dietary Niacin Intake and Mortality Stratified by Baseline Characteristics. **A** for all-cause mortality, **B** for cancer mortality. Adjusted for age, sex, race, bmi, education, smoking status, drinking, diabetes, aspirin use, physical activity, energy intake, protein intake, sugar, carbohydrate, total fat intake, Vit B1, VitB2, Cholesterol, fiber

Stratified analyses were conducted to determine if niacin intake and mortality differed by sex, age, BMI, diabetes, smoking, drinking, or vigorous activity. The results were also reliable, except for aspirin use, dietary niacin shows protective effects in other subgroups. There was no statistically significant interaction for all-cause mortality and cancer mortality (Fig. 3). We further investigated the association between niacin intake and mortality by stratifying niacin intake levels below and above the recommended level. And the conclusions were also stable (Supplementary Fig. 3).

## Discussion

Our study found that higher intake of dietary niacin was associated with lower risk of mortality from all-causes and cancer mortality. The consumption of niacin had a dose-effect relationship for all-cause mortality, but not for cancer mortality. This conclusion was verified by the data of supplemental niacin consumption. The results of our study are consistent with those of other recent cohort studies. According to Chen F et al [22], dietary nutrition is associated with a lower mortality rate, while supplement intake can be harmful in excess. A meta-analysis of 13 trials revealed a tendency towards a lower risk of cardiovascular mortality (RR = 0.91; 95%CI: 0.81–1.02) and coronary death (RR = 0.93; 95%CI: 0.78–1.10) with niacin treatment. But niacin and control arms did not differ in all-cause mortality rates (RR = 0.99; 95%CI: 0.88–1.12) [23]. Park SM et al. found that niacin may be beneficial to SCC. The HRs (95%CI) of skin cancer of total niacin intake for top vs. bottom quintiles were 0.80 (0.67, 0. 96) [8]. A study conducted by Surjana D et al. indicated that niacin reduces DNA damage and carcinogenesis in various cancers, including breast, colon, lung, and oral cancers [24]. The effects of which may reduce cancer metastases and recurrences, and improve survival rates. A new study on the relationship between niacin and cancer reveals that NAD is consumed as a substrate in the adenosine diphosphoribose (ADP-ribose) transfer reaction [25]. ADP-ribose is a post-translational modification of nuclear proteins in many eukaryotic cells and has been linked to many important cellular processes, particularly DNA repair and apoptosis [26]. NAD synthesizes ADP-ribose polymers in response to carcinogen-induced DNA damage [27]. It may explain why, unlike niacin dose-effect relationships with all-cause death, there was no plateau in cancer mortality.

## Strengths and limitations

The strengths of our study include novel finding of association between niacin intake and mortality in patients with cancer, the large sample size, reliable mortality status and long duration of follow-up time. Moreover, the conclusions of our study were internally verified. We accounted for complex survey design in our statistical analysis, which was representative of the non-institutionalized civilian population in the US.

The study has some limitations. First, it is an observational study, so residual confounding cannot be excluded. However, a number of covariates have been altered to reduce confounding. Additionally, dietary measurements in our study were derived from self-reported 24-h recalls and may be biased by recall. However, it is the most commonly used method for collecting dietary intake data, and it is carried out by trained interviewers [28, 29]. Third, cancer and cardiovascular disease diagnoses were based on questionnaire data in self-reported interviews, without standardized medical records. Nevertheless, all interviewers were well-trained and used a computer-aided personal interviewing system (CAPI) to reduce errors in data entry. Fourth, due to the limited number of deaths, detailed cancer information was lacking. Therefore, more large-scale studies are required, including clinical trials.

## Conclusions

The intake of dietary niacin is associated with lower rates of death from cancer and all-cause among cancer patients. Supplemental niacin intake improves cancer mortality survival but not all-cause mortality.

## Supplementary Information

**Additional file 1: Supplementary Fig. 1.** Dose-Response Relationship Between Niacin Intake and Mortality. A for cancer-specific mortality, B for all-cause mortality. Adjusted for age, sex, race, bmi, education, smoking status, drinking, diabetes, aspirin use, physical activity, energy intake, protein intake, sugar, carbohydrate, total fat intake, Vit B1, VitB2, Cholesterol, fiber.

**Additional file 2: Supplementary Fig. 2.** Histogram for Niacin Intake in Supplement Group And no Supplement Group.

**Additional file 3: Supplementary Fig. 3.** A Stratified Analysis of Niacin Intakes Below and Above the Recommended Levels. A for all-cause mortality, B for cancer mortality. Adjusted for age, sex, race, bmi, education, smoking status, drinking, diabetes, aspirin use, physical activity, energy intake, protein intake, sugar, carbohydrate, total fat intake, Vit B1, VitB2, Cholesterol, fiber.

## Data Availability

The CDC NHANES website provides access to the data collected as part of the survey (https://www.cdc.gov/nchs/nhanes).
