# Supplementary figures and images for: Association between niacin and mortality among patients with cancer in the NHANES retrospective cohort
Source: BMC Cancer. 2022 Nov 14;22:1173. doi: 10.1186/s12885-022-10265-4 (PMC9661743; doi:10.1186/s12885-022-10265-4)

**A**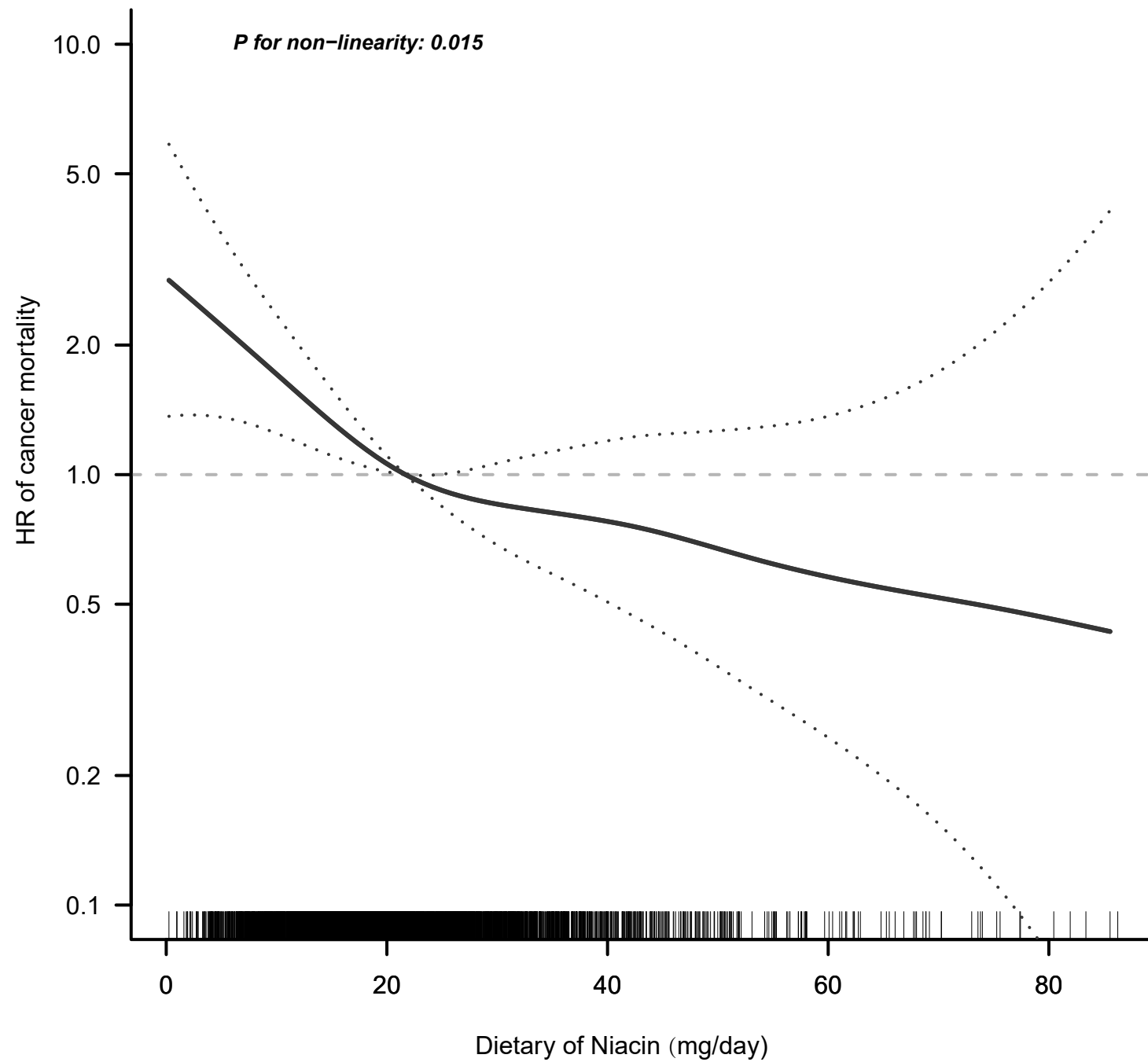**B**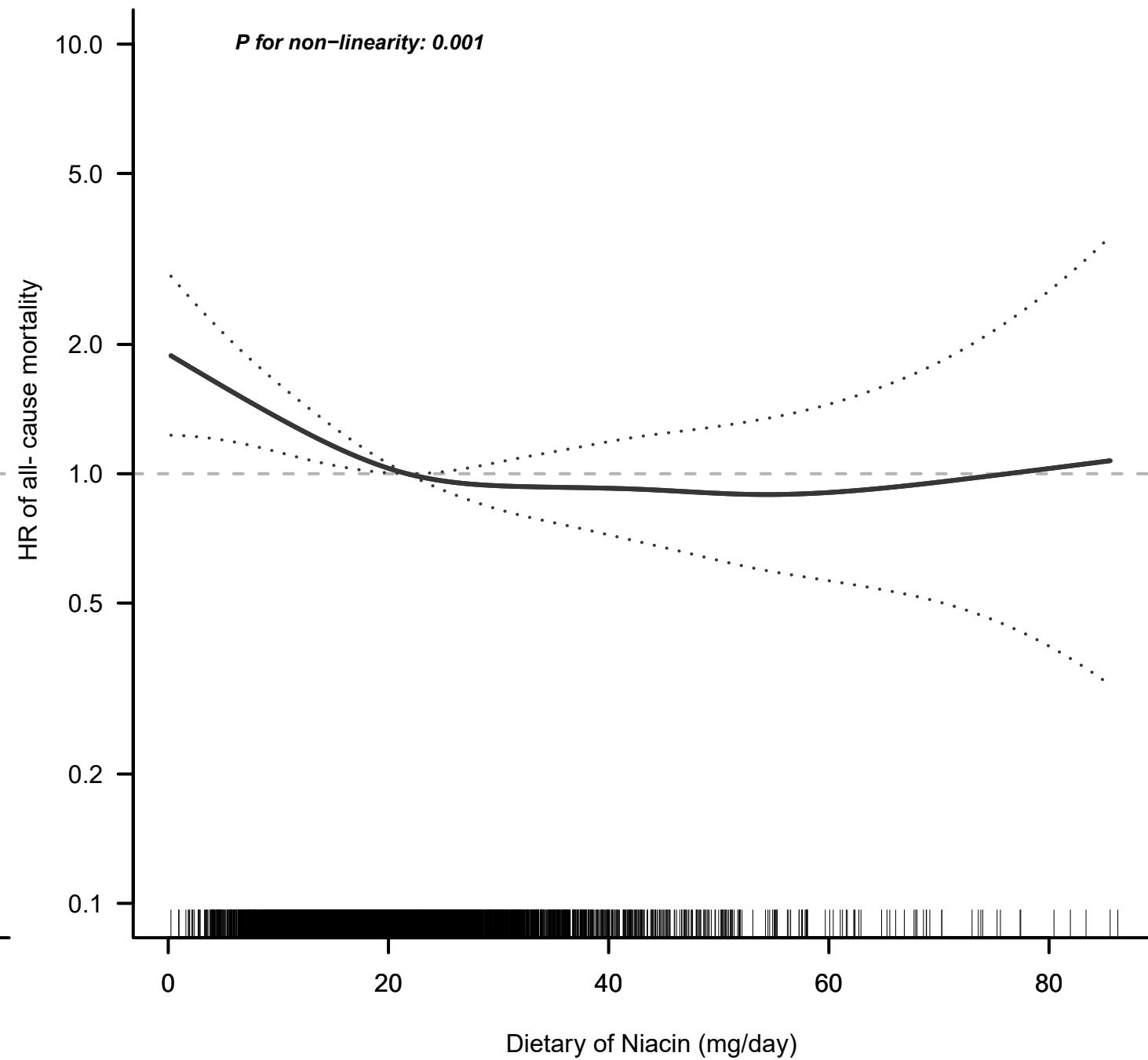

Supplement: Supplementary file 1 — Additional file 1: Supplementary Fig. 1. Dose-Response Relationship Between Niacin Intake and Mortality. A for cancer-specific mortality, B for all-cause mortality. Adjusted for age, sex, race, bmi, education, smoking status, drinking, diabetes, aspirin use, physical activity, energy intake, protein intake, sugar, carbohydrate, total fat intake, Vit B1, VitB2, Cholesterol, fiber. [file 12885_2022_10265_MOESM1_ESM.pdf]

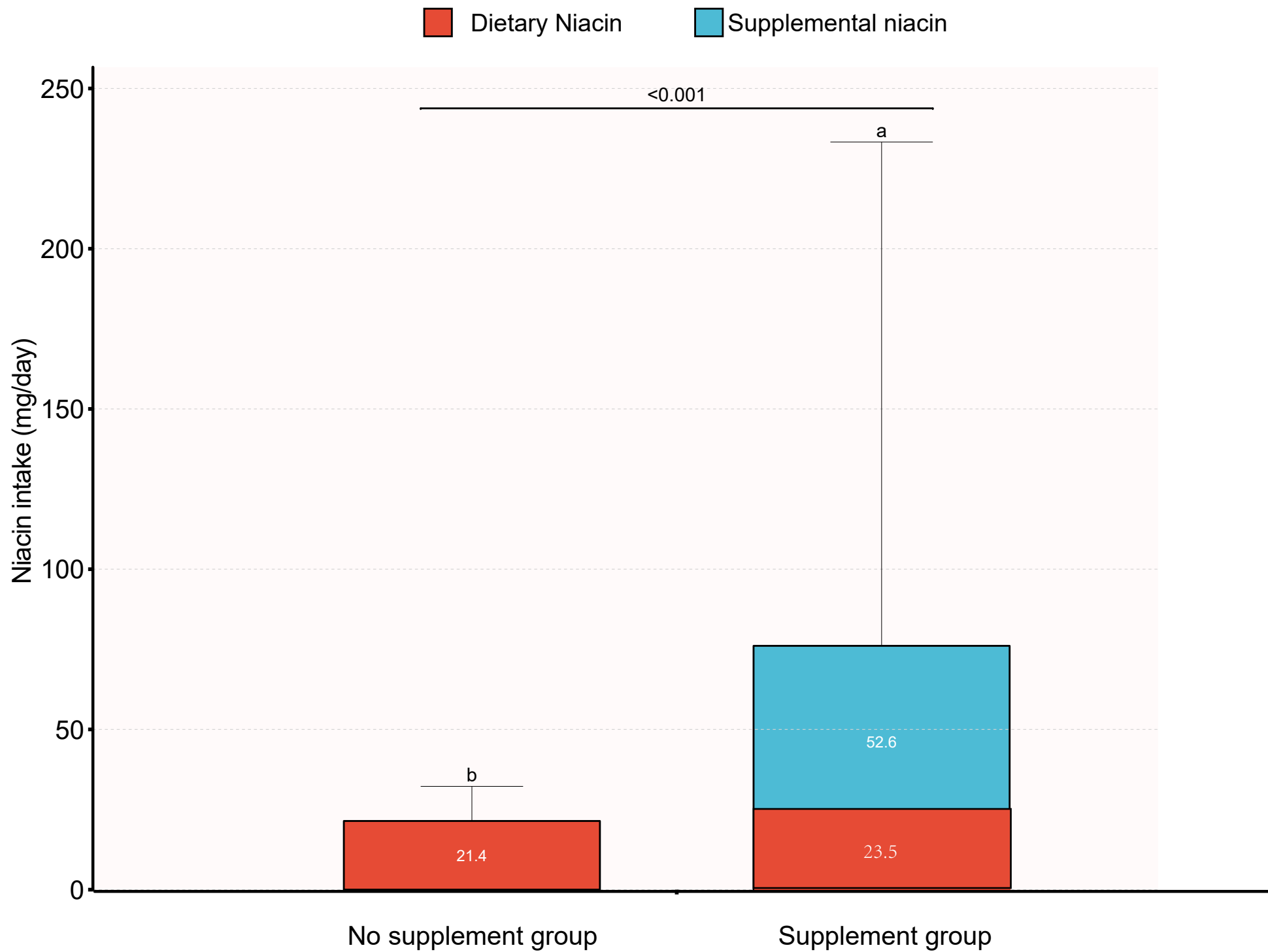

Supplement: Supplementary file 2 — Additional file 2: Supplementary Fig. 2. Histogram for Niacin Intake in Supplement Group And no Supplement Group. [file 12885_2022_10265_MOESM2_ESM.pdf]

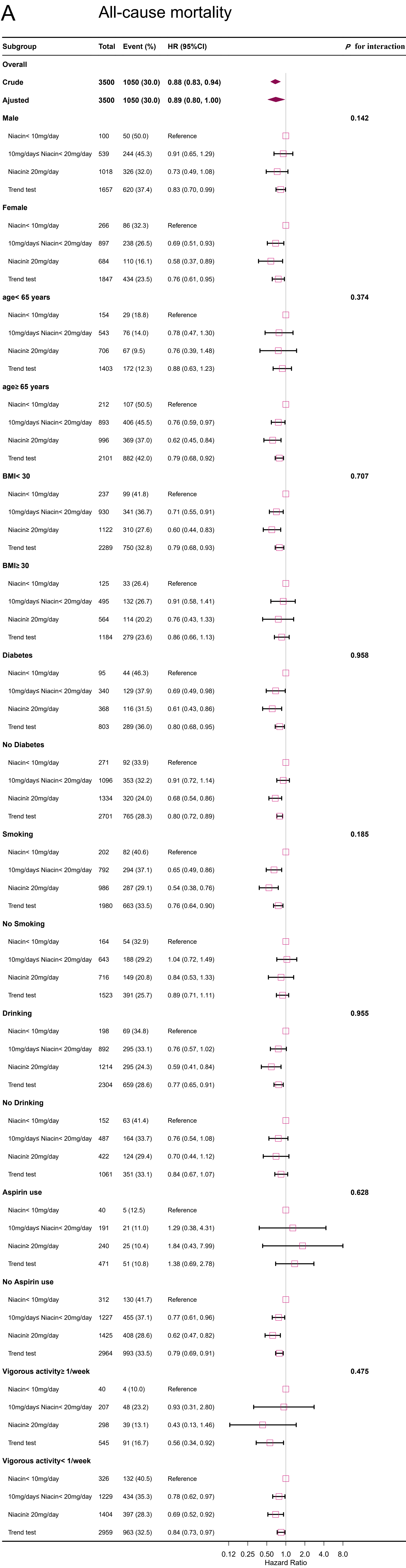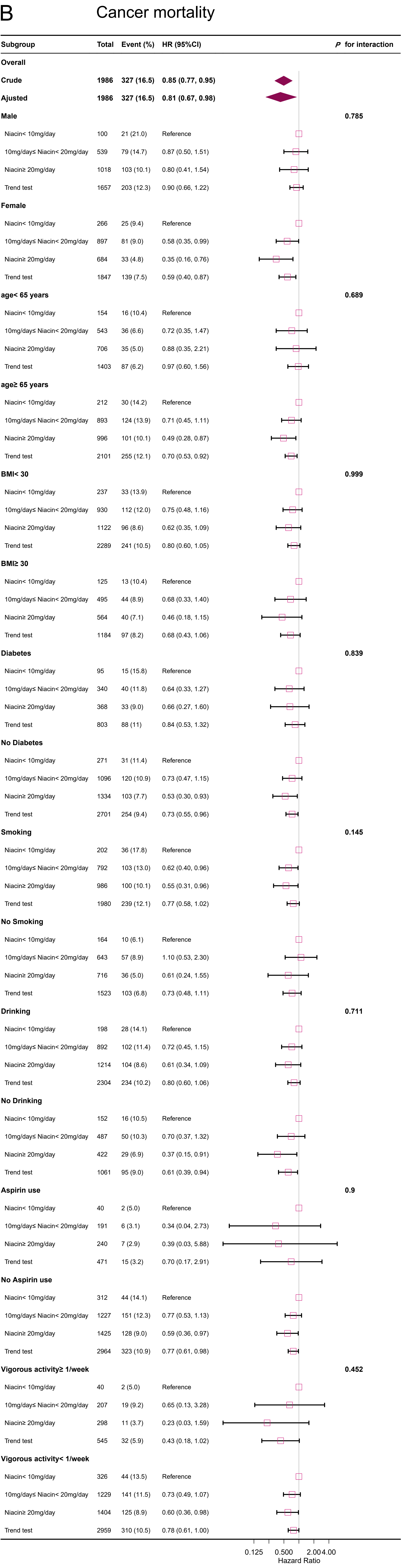

Supplement: Supplementary file 3 — Additional file 3: Supplementary Fig. 3. A Stratified Analysis of Niacin Intakes Below and Above the Recommended Levels. A for all-cause mortality, B for cancer mortality. Adjusted for age, sex, race, bmi, education, smoking status, drinking, diabetes, aspirin use, physical activity, energy intake, protein intake, sugar, carbohydrate, total fat intake, Vit B1, VitB2, Cholesterol, fiber. [file 12885_2022_10265_MOESM3_ESM.pdf]
